# Comparative Evaluation of Coronal Microleakage Associated with Different Intra-Orifice Barrier Protocols in Endodontically Treated Maxillary Anterior Teeth

**DOI:** 10.3390/jfb17100504

**Published:** 2026-10-06

**Authors:** Ceren Turan Gökduman, Esra Arılı Öztürk, Mediha Büyükgöze-Dindar

**Affiliations:** 1 Private Clinic, 39000 Kırklareli, Türkiye; crntrn15@gmail.com; 2 Private Clinic, 59000 Tekirdağ, Türkiye; 3 Department of Restorative Dentistry, Faculty of Dentistry, Trakya University, 22030 Edirne, Türkiye; medihabuyukgoze@trakya.edu.tr

**Keywords:** coronal microleakage, intra-orifice barrier, root canal treatment

## Abstract

Preventing coronal microleakage is crucial for long-term endodontic success; this ex vivo study compared the sealing performance of five intra-orifice barrier (IOB) material application protocols with that of a no-IOB control. Sixty maxillary anterior teeth were randomly assigned to six groups (*n* = 10): control, flowable composite, universal composite, resin-modified glass ionomer cement (RMGIC), high-viscosity bulk-fill composite, and flowable bulk-fill composite. Following endodontic treatment, 2 mm-thick barriers were placed in five groups. All specimens underwent 1000 thermocycles and 24 h immersion in 2% methylene blue. Linear dye penetration was measured at ×50 using stereomicroscopy and ImageJ. Data were analyzed using one-way ANOVA with Tukey’s HSD test (α = 0.05). Microleakage differed significantly among groups (F (5, 54) = 12.473; *p* < 0.001). The lowest mean microleakage was observed with the Estelite Sigma Quick (EQ) (1.85 ± 0.59 mm), followed by the Estelite Bulk Fill Flow (EF) (2.28 ± 0.91 mm) and Filtek One Bulk Fill Restorative (FR) (2.48 ± 1.18 mm). No statistically significant differences were detected among these materials; however, each demonstrated significantly less microleakage than the Tetric N-Flow (TNF), Ionoseal (IS), and no-IOB control (*p* < 0.05), among which no differences were detected (*p* > 0.05). Under the experimental conditions of this ex vivo study, the protocols involving the EQ and both bulk-fill resin composites (FR, EF) resulted in significantly lower coronal dye penetration than the protocols involving the TNF, IS and the no-IOB control.

## 1. Introduction

The long-term outcome of root canal treatment depends on effective microbial control within the root canal system and the subsequent maintenance of an adequate coronal seal [1]. Although thorough chemo-mechanical preparation and three-dimensional obturation are fundamental to successful endodontic therapy, preservation of the integrity of the definitive coronal restoration is equally critical for preventing microbial recontamination of the obturated canal system [2]. Disruption of the coronal seal as a consequence of marginal deterioration, recurrent caries, restorative or tooth fracture, or loss of the coronal restoration may establish a pathway for the penetration of microorganisms and their metabolic by-products into the root canal system, thereby compromising the biological conditions established during endodontic treatment and increasing the risk of secondary intraradicular infection [3,4]. Consequently, maintenance of an effective coronal seal represents an essential component of the long-term management of endodontically treated teeth, and the quality and integrity of the definitive coronal restoration have been identified as important determinants of treatment outcome [2].

The concept of an intra-orifice barrier (IOB) was introduced as an additional measure to reduce the risk of coronal contamination when the integrity of the definitive coronal restoration is compromised [3]. The technique involves the removal of a portion of the coronal gutta-percha and sealer from the canal orifice, followed by placement of a restorative material within the prepared space [5]. By establishing a supplementary barrier between the coronal restoration and the remaining root canal filling, an IOB may provide an additional level of protection against coronal microleakage and bacterial penetration [3]. Various materials, including glass-ionomer cements, resin-based composites, calcium silicate–based materials, amalgam, and zinc oxide–eugenol–based materials, have been investigated for this purpose [5,6].

The sealing performance of an intra-orifice barrier (IOB) is governed by the physicochemical, mechanical, and handling characteristics of the restorative material, as well as its interfacial interaction with dentin and the underlying root canal filling [5,7]. Adequate adaptation to the cavity walls, dimensional stability, resistance to functional stresses, effective adhesion to dentin, and compatibility with subsequent restorative procedures are considered important requirements for an IOB material [7]. Resin-based composites and resin-modified glass-ionomer cements (RMGICs) are among the materials most frequently evaluated [3]. The sealing behavior of resin composites is influenced by several material-dependent factors, including viscosity, filler loading, resin matrix composition and polymerization shrinkage [8]. Flowable composites generally exhibit lower viscosity, which may facilitate their adaptation to the restricted and potentially irregular geometry of the canal orifice [5,9]. However, formulations with relatively lower filler loading may exhibit greater volumetric polymerization shrinkage and comparatively reduced mechanical properties that may influence their interfacial integrity and resistance to microleakage [8]. RMGICs, in contrast, undergo both acid–base and resin polymerization reactions and exhibit an adhesive interaction with dental hard tissues [10]. Their relatively favorable handling characteristics, fluoride release, and ability to establish an adhesive interaction with dentin have contributed to their use as intra-orifice barrier materials [7]. Bulk-fill resin composites were developed to facilitate placement in greater increment thicknesses while maintaining an adequate degree of polymerization at increased depths [11]. As the distance from the curing-light tip increases, irradiance and curing depth may be reduced [12]. Bulk-fill composites are designed to achieve adequate curing at greater depths [11,13], although light attenuation within the material may still affect polymerization [14]. These factors may be relevant to IOB applications. Bulk-fill resin composites are also available in both flowable and high-viscosity formulations, which differ in filler loading, rheological properties, resin matrix composition, and polymerization kinetics [5,8,11]. However, despite the variety of materials investigated, no consensus has been reached regarding the optimal IOB material [3,4,5].

Previous in vitro investigations evaluating intra-orifice barriers have employed heterogeneous experimental protocols with respect to barrier thickness, restorative materials, aging conditions, and microleakage assessment methods, thereby limiting direct comparison among studies [3,5,7]. Consequently, direct comparisons among contemporary IOB materials under standardized experimental conditions remain limited [3,5].

Accordingly, the present study aimed to comparatively assess coronal microleakage associated with five intra-orifice barrier material–application protocols involving RMGIC, flowable resin composite, universal resin composite, high-viscosity bulk-fill resin composite, and flowable bulk-fill resin composite, relative to a no-IOB control in endodontically treated human maxillary anterior teeth following thermocycling. The null hypothesis was that no statistically significant differences in coronal microleakage would be detected among the six experimental groups.

## 2. Materials and Methods

### 2.1. Ethics Approval

Ethical approval for the use of extracted human teeth was obtained from the Trakya University Non-Interventional Scientific Research Ethics Committee (TUTF-GOBAEK 2026/344). The study was conducted in accordance with the ethical principles and institutional regulations applicable to research involving human biological materials. Written informed consent was obtained from each patient prior to tooth extraction, authorizing the use of the extracted teeth for scientific research purposes.

This ex vivo laboratory study was designed and reported in accordance with the PRILE 2021 guidelines for reporting laboratory studies in Endodontology [15].

### 2.2. Sample Size Estimation

An a priori sample size calculation was performed using G*Power software, version 3.1.9.7 (Heinrich Heine University, Düsseldorf, Germany). The primary outcome used for the sample size estimation was mean dye penetration (coronal microleakage). The effect-size estimate was derived from the findings reported by Jiang et al. [16]. Specifically, the positive and negative control groups were excluded, and the six material groups reported in that study were considered. For a six-group experimental design, an effect size of f = 0.513, an α level of 0.05, and a statistical power of 80% yielded a minimum required sample size of 60 specimens. Accordingly, 10 specimens were allocated to each experimental group.

### 2.3. Sample Selection and Preparation

A total of 60 extracted human maxillary anterior teeth were included in this ex vivo study. The teeth had been extracted for periodontal, orthodontic, or prosthodontic reasons and were obtained in accordance with the approved ethical protocol. Maxillary central incisors, lateral incisors, and canines with fully developed root apices, a single root, and a single root canal were considered eligible for inclusion. Teeth with cracks, fractures, root resorption, previous endodontic treatment, extensive caries, root surface restorations, or other structural abnormalities were excluded. Teeth exhibiting pronounced root curvature were also excluded to minimize anatomical variability and facilitate standardized root canal instrumentation. Periapical radiographs were obtained in the buccolingual and mesiodistal directions to confirm the presence of a single root canal and to evaluate root and canal morphology.

Following extraction, residual soft tissues and periodontal ligament remnants were removed from the external root surfaces using periodontal curettes and ultrasonic instruments. The teeth were thoroughly rinsed with distilled water and stored in 0.5% chloramine-T solution at room temperature until use. The storage solution was renewed periodically to minimize the risk of microbial contamination. Before specimen preparation, the teeth were removed from the chloramine-T solution and thoroughly rinsed with distilled water; they were not subsequently stored in distilled water.

Prior to randomization, the radiographic images were reviewed to identify specimens with comparable root and canal morphology. Teeth exhibiting marked anatomical variations, including unusually wide, narrow, flattened, or irregular canals, were excluded. Specimens with comparable anatomical characteristics were selected for inclusion before randomization to minimize anatomical variability among the study specimens.

The crowns were sectioned using a diamond disc under continuous water cooling, and the root length was standardized to 16 mm. Canal patency was initially established using a size #10 K-file (Dentsply Tulsa Dental Specialties, Tulsa, OK, USA). The file was advanced until its tip was visible at the apical foramen, and the working length was established 1 mm short of this measurement. Thus, a standardized working length of 15 mm was established for all specimens.

Root canal preparation was performed using the VDW.ROTATE nickel-titanium rotary system (VDW GmbH, Munich, Germany) according to the manufacturer’s recommendations, and all canals were prepared to a final apical size of #40/.04. During instrumentation, 2 mL of 2.5% sodium hypochlorite solution (Microvem, Sakarya, Türkiye) was delivered after each rotary instrument. Apical patency was maintained using a size #10 K-file.

After completion of canal preparation, 5 mL of 17% ethylenediaminetetraacetic acid (EDTA) (Promida, Eskişehir, Türkiye) was applied for 1 min to remove the smear layer. The canals were subsequently rinsed with 5 mL of 2.5% sodium hypochlorite solution. Following final irrigation, the canals were dried with sterile paper points. A #40/.04 gutta-percha cone was fitted to the established working length, and its adaptation was confirmed by the presence of tug-back. A premixed calcium silicate–based bioceramic root canal sealer (One-Fil; Mediclus Co., Ltd., Cheongju, Republic of Korea) was injected directly into the root canal using the manufacturer’s intracanal tip until the sealer became visible at the canal orifice. The fitted #40/.04 gutta-percha cone was then inserted to the full working length, and obturation was completed using the single-cone technique. Excess gutta-percha was removed at the canal orifice using a heated instrument. Residual sealer on the canal orifice and adjacent dentinal surfaces was removed using cotton pellets moistened with alcohol [17]. All root canal preparation and obturation procedures were performed by the same operator (C.T.G.)

### 2.4. Standardization of Intra-Orifice Cavities

Following root canal filling, the coronal portion of the root canal filling material was removed using a standardized sequential preparation technique. First, the canal orifices were uniformly enlarged using a size #4 Gates–Glidden drill (1.1 mm diameter) [18]. Subsequently, the intra-orifice cavities were enlarged using a fissure bur to achieve a standardized 2 mm cavity [19]. A reference mark was placed on the fissure bur at 2 mm from its tip to standardize the cavity depth among specimens, and the depth was subsequently verified using a periodontal probe. The cavity dimensions were kept as similar as possible among specimens using the same preparation sequence and instruments. However, because the final cavity geometry was influenced by the anatomical configuration of each tooth, complete standardization of the buccolingual and mesiodistal dimensions could not be achieved.

The cavity surfaces were subsequently air-abraded using 53 µm aluminum oxide particles with an AquaCare unit (Velopex International, London, UK) at a pressure of 4 bar for 5 s [20]. The device tip was positioned 5 mm from the dentin surface at a 45° inclination, and water-assisted airborne-particle abrasion was used. The entire internal cavity surface was treated using a standardized procedure, and all air-abrasion procedures were performed by the same operator (M.B.D.). Following air abrasion, the cavity surfaces were thoroughly rinsed with water to remove residual abrasive particles and debris. The specimens were stored at 37 °C and 100% relative humidity for 7 days to allow adequate setting of the calcium silicate–based sealer.

### 2.5. Intra-Orifice Barrier Application

Following preparation of the standardized intra-orifice cavities, the 60 specimens were randomly allocated to six experimental groups (*n* = 10 per group) using a computer-generated randomization sequence obtained from Randomizer.org. Except for the control and RMGIC groups, Tokuyama Universal Bond (Tokuyama Dental Corp., Tokyo, Japan) was applied to the prepared cavity surfaces according to the manufacturer’s instructions and gently air-dried before placement of the respective barrier material. The materials evaluated in the present study, together with their corresponding compositions and manufacturers, are presented in Table 1.

The experimental groups were as follows:

Group 1—No-IOB Control: No intra-orifice barrier material was applied.

Group 2—Flowable composite: Tetric N-Flow (TNF; Ivoclar Vivadent, Schaan, Liechtenstein).

Group 3—Universal composite: Estelite Sigma Quick (EQ; Tokuyama Dental Corp., Tokyo, Japan).

Group 4—RMGIC: Ionoseal (IS; VOCO GmbH, Cuxhaven, Germany).

Group 5—High-viscosity bulk-fill composite: Filtek One Bulk Fill Restorative (FR; 3M ESPE, St. Paul, MN, USA).

Group 6—Flowable bulk-fill composite: Estelite Bulk Fill Flow (EF; Tokuyama Dental Corp., Tokyo, Japan).

The materials evaluated in the present study were selected to enable a comparative assessment of commercially available restorative materials with different material characteristics that may influence their performance as IOBs. This selection allowed comparison of materials differing in material type, viscosity, filler characteristics, and conventional versus bulk-fill formulation.

The conventional universal and flowable resin composites were included to compare two commonly used resin-based formulations with different viscosities and filler characteristics. Ionoseal was selected as a resin-modified glass-ionomer material with an established adhesive interaction with dentin and clinical applicability in confined restorative spaces. The two bulk-fill resin composites were selected to evaluate the sealing performance of different bulk-fill formulations, specifically a flowable and a high-viscosity formulation, at a standardized IOB thickness. Thus, the selected materials were intended to provide variation in formulation and handling characteristics while allowing comparison under a standardized experimental protocol.

All barrier materials were placed to a standardized thickness of 2 mm and polymerized using an Elipar DeepCure-S LED light-curing unit (3M ESPE, St. Paul, MN, USA). The light-curing unit was checked using an SDI radiometer (Southern Dental Industries, Bayswater, VIC, Australia) before specimen preparation to verify the light output. The irradiance specified by the manufacturer for the Elipar DeepCure-S is 1470 mW/cm^2^. All materials were light-cured for 20 s using a standardized curing protocol. Although the manufacturers’ recommended curing times differ among the tested materials, a uniform 20 s exposure was intentionally selected to standardize the energy delivery conditions across all experimental groups. This approach ensured that the curing time was not an additional experimental variable and provided an exposure duration at least as long as the recommended curing time for the tested materials under comparable high-intensity LED conditions. The light-curing unit was positioned perpendicular to and in direct contact with the coronal surface of the restorative material throughout polymerization.

### 2.6. Thermocycling and Dye Penetration

Following completion of the restorative procedures, the specimens were stored in distilled water at 37 °C for 24 h. Finishing and polishing of the restored surfaces were performed using Sof-Lex discs (3M ESPE, St. Paul, MN, USA) in the sequence of coarse, medium, fine, and extra-fine grits, with each disc applied for 10 s under water cooling. The specimens were then subjected to 1000 thermocycles (Thermocycler THE 1100, SD Mechatronik, Feldkirchen-Westerham, Germany) between 5 °C and 55 °C, with a dwell time of 30s in each bath and a transfer time of 10s.

After thermocycling, the external surfaces of all specimens were coated with three layers of nail varnish, except for a 1 mm-wide area surrounding the margins of the IOB. A fresh 2% methylene blue solution (KimyaLab, Istanbul, Türkiye) was prepared by dissolving 2 g of methylene blue powder in 100 mL of distilled water. The specimens were immersed in a 2% methylene blue solution at 37 °C for 24 h. Following dye exposure, the specimens were thoroughly rinsed with distilled water to remove residual dye from the external surfaces.

Before sectioning, the specimens were embedded in self-cure acrylic resin blocks to provide stability. Each specimen was longitudinally sectioned through the center of the intra-orifice barrier in the buccolingual direction using a diamond disc under continuous water cooling, thereby dividing each tooth into two halves and providing two opposing sectioned surfaces for microleakage assessment. The sectioning plane was standardized by positioning the cut through the center of the intra-orifice cavity and maintaining the same buccolingual orientation for all specimens.

### 2.7. Microleakage Assessment

Coronal microleakage was evaluated using a stereomicroscope (SZ61; Olympus Corp., Tokyo, Japan) at ×50 magnification. Digital images of the sectioned specimens were acquired and transferred to ImageJ software (Version 1.53; National Institutes of Health, Bethesda, MD, USA). The images were calibrated using a reference scale before measurements were performed.

Microleakage was quantified as the linear extent of methylene blue penetration, measured in millimeters in the apical direction from the most coronal margin of the standardized 2 mm intra-orifice cavity to the most apical extent of visible dye penetration. The same measurement path and endpoint definition were applied to both the IOB and no-IOB control groups. In the no-IOB control group, the standardized 2 mm coronal space remained unfilled; therefore, the unfilled space constituted part of the measured dye-penetration path, and any additional penetration beyond this space was measured along the underlying root-filling interface.

Each tooth was divided into two halves by a single central buccolingual section, and the maximum apical extent of dye penetration was measured separately on the exposed sectioned surface of each half. The two measurements obtained from the same specimen were then averaged to generate a single specimen-level microleakage value, which was used for statistical analysis. Thus, each tooth contributed one value to the statistical analysis and was considered the statistical unit.

All measurements were performed by a trained examiner who was blinded to the experimental group assignments.

### 2.8. Statistical Analysis

Statistical analyses were performed using IBM SPSS Statistics for Windows (Version 31.0; IBM Corp., Armonk, NY, USA). To evaluate intra-examiner reliability, 20% of the specimens were randomly selected and re-measured by the same examiner after a two-week interval. Intra-examiner agreement was assessed using the intraclass correlation coefficient (ICC).

Data are presented as mean ± standard deviation (SD).

Data normality and homogeneity of variances were assessed using the Shapiro–Wilk and Levene’s tests, respectively. Differences among the experimental groups were analyzed using one-way analysis of variance (ANOVA), followed by Tukey’s honestly significant difference (HSD) post hoc test for pairwise comparisons. Statistical significance was established at *p* < 0.05.

## 3. Results

**Figure 1:**
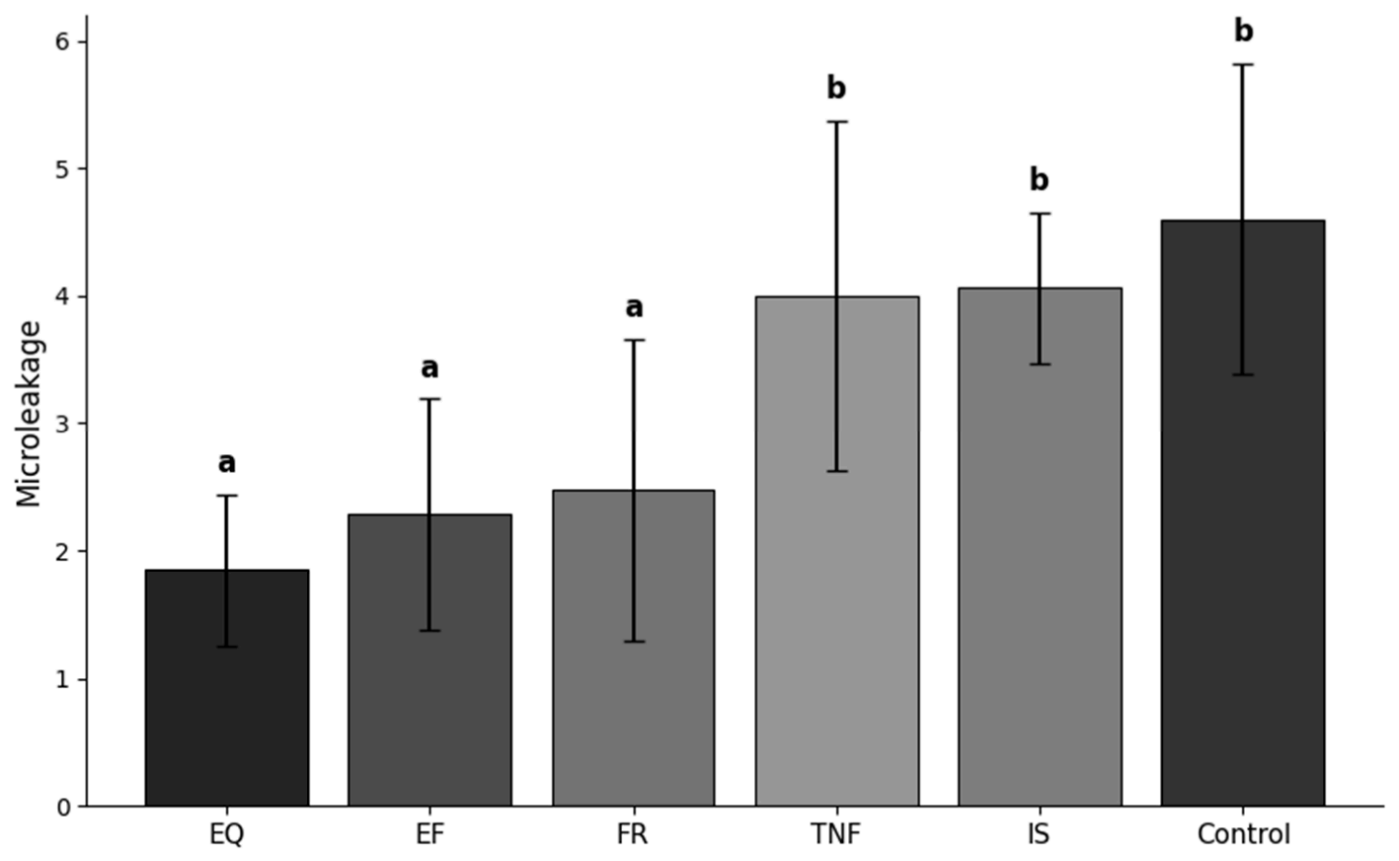
Mean microleakage values of the experimental groups. Error bars represent standard deviations. Groups sharing the same superscript letter were not significantly different according to Tukey’s honestly significant difference (HSD) post hoc test (α = 0.05). EQ, Estelite Sigma Quick; EF, Estelite Bulk Fill Flow; FR, Filtek One Bulk Fill Restorative; TNF, Tetric N-Flow; IS, Ionoseal; Control, no-IOB control.

**Figure 2:**
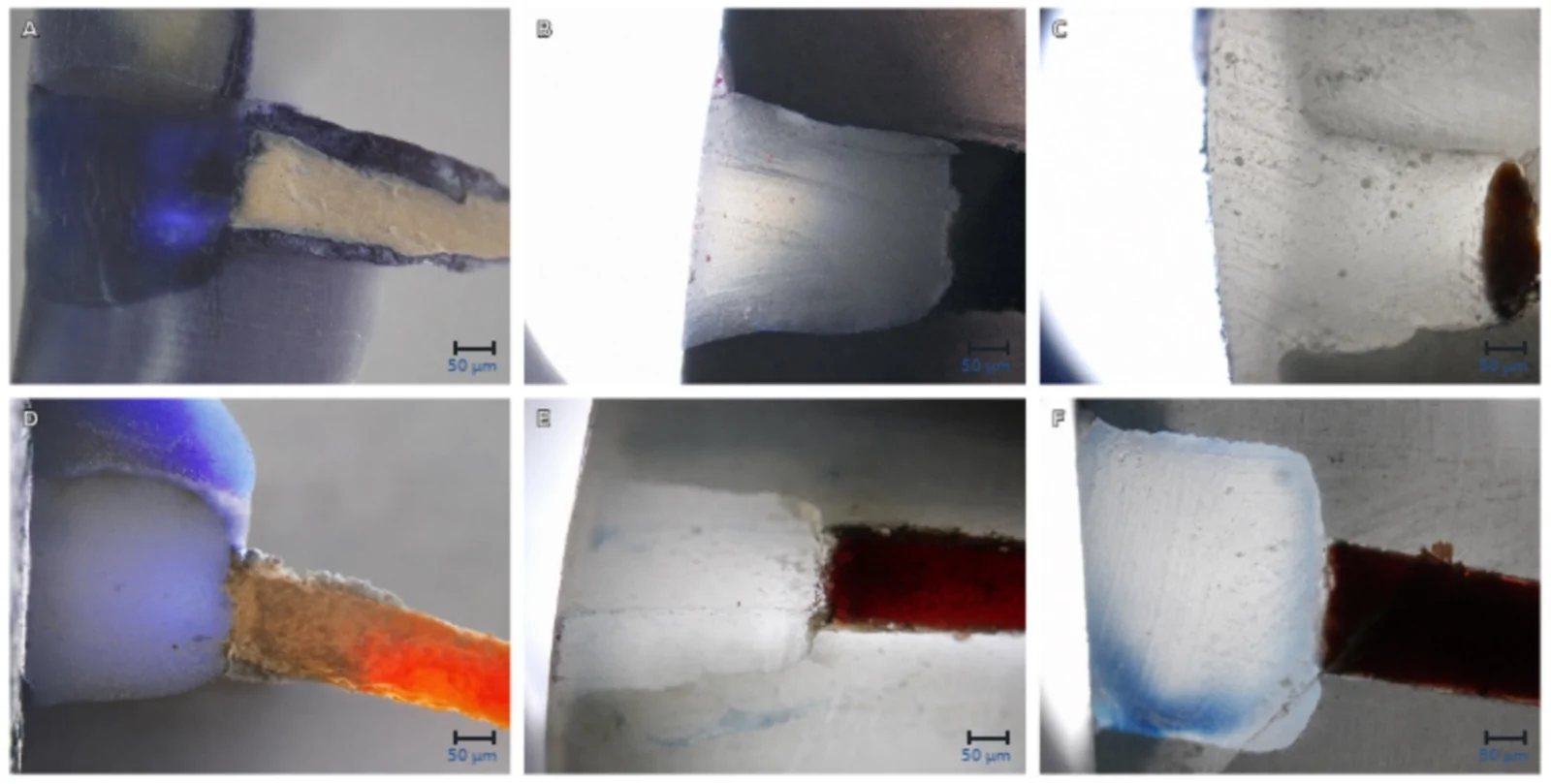
Representative stereomicroscopic images of dye penetration in the experimental groups. (**A**) no intra-orifice barrier control group, (**B**) Tetric N-Flow (TNF), (**C**) Estelite Sigma Quick (EQ), (**D**) Ionoseal (IS), (**E**) Filtek One Bulk Fill Restorative (FR), (**F**) Estelite Bulk Fill Flow (EF), Scale bar = 50 µm.

The descriptive statistics for microleakage are presented in Table 2, and the corresponding graphical representation is shown in Figure 1. Representative stereomicroscopic images of dye penetration for the six experimental groups are shown in Figure 2. Intra-examiner reliability was assessed by repeating the measurements in 20% of the specimens. Intra-examiner reliability was assessed using a two-way mixed-effects intraclass correlation coefficient (ICC) based on single measures and absolute agreement. Excellent intra-examiner reliability was observed at 0.998 (95% CI: 0.995–0.999; *p* < 0.001).

One-way ANOVA demonstrated a statistically significant difference in microleakage among the experimental groups, F(5,54) = 12.473, *p* = 4.61 × 10^−8^, with a large overall effect size (η^2^ = 0.536).

The EQ group exhibited the lowest mean microleakage value (1.85 ± 0.59 mm), followed by the EF group (2.28 ± 0.91 mm) and the FR group (2.48 ± 1.18 mm). The highest mean microleakage value was observed in the no-IOB control (4.60 ± 1.22 mm), whereas the TNF and IS groups demonstrated mean values of 4.00 ± 1.37 mm and 4.06 ± 0.59 mm, respectively.

Tukey’s HSD post hoc analysis showed that the TNF group had significantly greater microleakage than the EF group (mean difference [MD] = 1.71 mm, 95% CI: 0.36–3.06, *p* = 0.006), FR group (MD = 1.51 mm, 95% CI: 0.16–2.86, *p* = 0.019), and EQ group (MD = 2.15 mm, 95% CI: 0.80–3.50, *p* < 0.001). The EF group demonstrated significantly lower microleakage than the IS group (MD = −1.78 mm, 95% CI: −3.13 to −0.42, *p* = 0.004) and the no-IOB control group (MD = −2.31 mm, 95% CI: −3.66 to −0.96, *p* < 0.001). Similarly, the FR group exhibited significantly lower microleakage than the IS group (MD = −1.58 mm, 95% CI: −2.93 to −0.23, *p* = 0.013) and the no-IOB control group (MD = −2.11 mm, 95% CI: −3.47 to −0.76, *p* < 0.001). The EQ group showed significantly lower microleakage than the IS group (MD = −2.21 mm, 95% CI: −3.56 to −0.86, *p* < 0.001) and the no-IOB control group (MD = −2.75 mm, 95% CI: −4.10 to −1.40, *p* < 0.001).

No statistically significant differences were observed among the EQ, EF, and FR groups (all *p* > 0.05). Likewise, no significant differences were detected between TNF and IS (*p* = 1.000), TNF and no-IOB control (*p* = 0.776), EF and FR (*p* = 0.998), EF and EQ (*p* = 0.928), or FR and EQ (*p* = 0.731). Thus, the Tukey HSD analysis identified two statistically homogeneous subsets: EQ, EF, and FR, and TNF, IS, and no-IOB control (Table 2 and Figure 1).

## 4. Discussion

The present study evaluated the coronal microleakage of five contemporary intra-orifice barrier IOB materials with different compositional and rheological characteristics and compared their performance with that of a no-IOB control following thermocycling. The principal finding was that coronal microleakage differed significantly among the evaluated IOB material–application protocols. The EQ, EF, and FR groups demonstrated significantly lower microleakage than the TNF, IS, and control groups, whereas no significant differences were detected within either of these two subsets. Thus, the null hypothesis was rejected. These findings indicate that, under the experimental conditions employed, coronal microleakage differed among the specific IOB materials evaluated. The EQ, EF, and FR groups demonstrated lower microleakage than the TNF, IS, and no-barrier control groups. Because only selected commercial formulations were investigated, these findings should not be interpreted as evidence of superiority or inferiority of the corresponding material categories as a whole.

The lower microleakage observed for EQ, EF, and FR compared with the no-IOB control is broadly consistent with previous evidence supporting the potential role of an additional barrier at the canal orifice [5]. Araújo et al., in a systematic review and meta-analysis of in vitro studies, reported a significant reduction in microleakage following IOB placement compared with root-filled teeth without an additional barrier [5]. However, substantial heterogeneity was observed among the included studies with respect to tooth type, barrier material, barrier depth, obturation technique, aging protocol, and leakage assessment method [3,5]. Similarly, Chen et al. reported that GIC, RMGIC, composite resin, MTA, and Cavit used as orifice barriers reduced bacterial microleakage compared with root canal fillings without an additional barrier, although no consistent superiority among GIC, RMGIC, composite resin, and MTA was demonstrated [3]. The present findings are therefore in agreement with the broader evidence that an additional material placed at the canal orifice can influence resistance to coronal leakage, while further demonstrating differences among contemporary resin-based formulations.

The methodological approach used to assess microleakage should also be considered when interpreting the present findings. Methylene blue dye penetration was selected because of its established use in laboratory microleakage models, relative technical simplicity, cost-effectiveness, and high sensitivity resulting from the low molecular weight and substantial penetration capacity of the dye [21,22,23]. In the present study, dye penetration was assessed quantitatively rather than by a qualitative or ordinal scoring system. Penetration was directly visualized on calibrated digital images and measured as a continuous linear distance in millimeters using ImageJ, allowing the apical extent of dye penetration along the sectioned interface to be determined.

Alternative microleakage methods provide different and potentially complementary information [21]. Dye extraction enables quantitative determination of the amount of dye that has penetrated the specimen but does not directly demonstrate the spatial extent of penetration along the interface [22]. Fluorescent dye assessment combined with confocal laser scanning microscopy may provide more detailed visualization of dye penetration and overcome some of the limitations associated with conventional section-based assessment [24]. Bacterial leakage models, including two-chamber systems, may provide greater biological relevance by directly assessing microbial penetration; however, their findings may also be influenced by methodological factors such as the microorganism selected, experimental configuration, and duration of observation [25]. Therefore, these approaches assess different aspects of leakage and should be regarded as complementary rather than directly interchangeable methods [21,22].

An important distinction should be made when interpreting the present no-IOB control group. No intra-orifice barrier material was placed in the control specimens following removal of the coronal 2 mm of the root canal filling. Thus, the control represented a no-IOB condition rather than a tooth restored with an adequately sealed definitive coronal restoration. The significantly greater microleakage observed in the control compared with EQ, EF, and FR therefore demonstrates that these materials provided an additional barrier against dye penetration under the experimental conditions. However, this comparison cannot establish whether an IOB provides an incremental sealing benefit when a clinically adequate definitive coronal restoration is subsequently placed.

This distinction is particularly relevant to the clinical rationale for IOBs. An IOB is not intended to replace the definitive coronal restoration; rather, it is proposed as a supplementary barrier that may provide additional protection if the definitive restoration subsequently becomes compromised, fractured, dislodged, or lost [26]. Therefore, the present results should not be interpreted as demonstrating that IOB placement is mandatory in every endodontically treated tooth with a well-adapted definitive restoration. Instead, they indicate that when an IOB is selected as an adjunctive measure, its sealing performance is dependent on the material used.

The EQ group exhibited the lowest numerical mean microleakage (1.85 ± 0.59 mm); however, this value was not statistically different from those of EF (2.28 ± 0.91 mm) and FR (2.48 ± 1.18 mm). Therefore, the present findings should not be interpreted as evidence of superior sealing performance of EQ over the bulk-fill materials. No statistically significant differences in microleakage were detected among the three materials. Previous studies have highlighted the importance of material composition and adhesive interaction with dentin in the sealing behavior of resin composites [16]. Estelite Sigma Quick contains supra-nano spherical filler particles with an average particle size of approximately 200 nm, and reported to have favorable polymerization shrinkage characteristics [27]. These material characteristics and the adhesive protocol may be relevant to sealing behavior; however, their contribution to the relatively low microleakage observed for EQ cannot be determined from the present findings. This is because polymerization shrinkage, degree of conversion, and interfacial adaptation were not independently evaluated in the present study.

An important consideration when interpreting the performance of EQ is the standardized 2 mm IOB thickness used in the present study. Barrier depths between 1 and 4 mm have been investigated, with 2–3 mm being among the most frequently evaluated configurations [3]. A 2 mm barrier was selected in the present study to provide a standardized experimental geometry while limiting the risk of excessive removal of coronal root filling material and potential procedural complications associated with greater barrier depths. However, the clinical configuration of an intra-orifice cavity is considerably more variable than this standardized model [28]. The effective distance between the curing-light tip and the deeper portion of the restorative material may exceed the nominal 2 mm barrier thickness depending on canal anatomy, access cavity design, tooth position, remaining coronal tooth structure, and the angulation and accessibility of the curing-unit tip [29]. Because irradiance decreases as the distance from the light source increases, these factors may adversely affect polymerization of conventional light-cured resin composites [12]. Consequently, the favorable microleakage observed for EQ in the present study should not necessarily be extrapolated to deeper or less accessible clinical configurations.

The absence of a statistically significant difference between EF and FR is particularly relevant to the rationale for investigating bulk-fill resin composites as IOB materials. EF is a flowable bulk-fill formulation, whereas FR is a high-viscosity bulk-fill restorative composite. The lower viscosity of EF may facilitate adaptation to the restricted geometry of the intra-orifice cavity, whereas the higher filler loading and viscosity of FR may contribute to dimensional stability and mechanical resistance [30]. Despite these differences, neither material demonstrated significantly different microleakage from the other. No statistically significant differences were detected between EF and FR under the experimental conditions; however, it does not establish whether their specific rheological or compositional characteristics contributed to this outcome.

The use of bulk-fill composites as IOB materials is of particular interest because these materials were developed to achieve adequate polymerization at greater increment thicknesses [11]. Placement of an IOB within the canal orifice inherently increases the distance between the curing-light source and the deeper regions of the restorative material, potentially reducing the radiant energy delivered to these areas [13]. In the present study, no statistically significant differences in microleakage were detected among EF, FR, and EQ under the standardized 2 mm configuration evaluated. However, because depth of cure and degree of conversion were not directly assessed, this finding cannot be attributed to the polymerization characteristics of the bulk-fill materials. Moreover, the curing protocol represented a controlled laboratory condition in which the curing unit was positioned perpendicular to and as close as possible to the coronal surface of the material. Such conditions may be more favorable than those encountered clinically. Therefore, the absence of a statistically significant difference between EF and FR should be interpreted cautiously, particularly when considering situations involving greater curing distances or unfavorable light-curing angulations.

The relatively favorable performance of the bulk-fill materials may also be related to their polymerization shrinkage and shrinkage-stress characteristics [31]. Polymerization shrinkage stress generated at the bonded interface is an important determinant of marginal integrity, and differences in polymerization behavior and internal marginal adaptation have been reported among conventional and bulk-fill resin composites [31]. A lower degree of interfacial stress development may contribute to preservation of marginal integrity and, consequently, reduced microleakage [31]. Nevertheless, because polymerization shrinkage, shrinkage stress, degree of conversion, and depth-dependent mechanical properties were not directly assessed in the present study, a causal relationship between these properties and the observed microleakage values cannot be established.

In contrast, TNF demonstrated significantly greater microleakage than EQ, EF, and FR, although its mean microleakage was numerically lower than that of the control. The relatively higher microleakage observed with TNF may reflect differences in its flowable formulation and comparatively lower filler loading; however, these material characteristics were not directly evaluated as determinants of microleakage in the present study. The low viscosity of flowable composites may facilitate adaptation to irregular cavity walls; however, the reduced filler concentration required to achieve this rheological behavior may increase the relative proportion of polymerizable resin matrix and influence polymerization shrinkage [9,32]. Polymerization shrinkage may generate interfacial stresses that compromise marginal integrity, particularly in a confined cavity configuration [31]. Nevertheless, filler loading, polymerization shrinkage, shrinkage stress, and interfacial adaptation were not directly evaluated in the present study. Thus, although the flowable consistency of TNF may theoretically favor adaptation, the present findings do not allow its higher microleakage to be attributed to polymerization-related or other material properties.

The adhesive protocol also warrants consideration. A universal adhesive was applied to the cavity surfaces before placement of the resin-based barrier materials. Standardization of the adhesive protocol minimized variability associated with the use of different adhesive systems and provided a consistent bonding procedure for the resin composite groups. It should also be considered that EQ and EF were used with an adhesive from the same manufacturer, and compatibility within a restorative system may potentially influence sealing performance. However, the present study was not designed to isolate the effect of manufacturer matching, and FR, which was produced by a different manufacturer from the adhesive, also demonstrated low microleakage. Therefore, the performance observed for EQ and EF cannot be attributed specifically to same-manufacturer adhesive–composite compatibility. Nevertheless, the measured microleakage represents the performance of the material–adhesive combinations rather than the restorative materials independently. In addition, the adhesive protocol was not applied to the RMGIC group, reflecting the different bonding mechanisms and manufacturer’s application protocols of the materials. Consequently, the differences between the resin-based and RMGIC groups should be interpreted within the context of their respective clinical application protocols.

Several limitations of the present study should be acknowledged. First, the ex vivo experimental design does not fully reproduce the complex biological, mechanical, thermal, and chemical conditions encountered clinically. Although thermocycling was employed to simulate repeated thermal stresses at the tooth–restoration interface, factors such as occlusal loading, masticatory fatigue, moisture fluctuations, enzymatic degradation, and bacterial biofilm exposure were not incorporated into the experimental model. The 1000-cycle protocol represented a standardized laboratory aging procedure rather than a simulation of a specific duration of clinical service. The relatively limited number of thermocycles should therefore be considered when interpreting the findings, as longer-term thermal aging may produce different interfacial effects. Consequently, the present findings should be interpreted as material performance under controlled laboratory conditions rather than as direct evidence of long-term clinical behavior.

Second, the study was restricted to human maxillary anterior teeth with relatively standardized root and canal morphology. Teeth with pronounced root curvature and marked anatomical variations were excluded to minimize experimental variability and facilitate standardized instrumentation, obturation, intra-orifice preparation, and light curing. Although this approach enhances internal validity, it limits the generalizability of the findings to posterior teeth, which may present greater anatomical complexity, different access cavity configurations, and less favorable conditions for light curing. In addition, specimens were not stratified according to tooth type, and the distribution of maxillary central incisors, lateral incisors, and canines among the experimental groups was not recorded. Therefore, potential differences in root and canal dimensions among these tooth types may have introduced additional variability. Another limitation is that the experimental groups represented complete material–application protocols rather than the isolated effects of the restorative materials. In particular, differences in adhesive application among the tested protocols may have contributed to the observed microleakage and therefore preclude attribution of the findings solely to the intrinsic properties of the materials. Furthermore, each material category was represented by a specific commercial product; consequently, the findings should be interpreted as product- and protocol-specific and should not be generalized to all materials within the corresponding broader material categories.

Third, despite providing quantitative measurement of the extent of dye penetration, the section-based method used in the present study has inherent limitations. Dye penetration provides an indirect assessment of interfacial leakage and does not reproduce the complex mechanisms of bacterial ingress or fluid movement through the root canal system [33]. Moreover, the extent of dye penetration may be influenced by the physicochemical properties of the tracer, specimen preparation, sectioning procedures, and the criteria used for microscopic evaluation [34]. In particular, methylene blue may undergo photochemical changes upon prolonged exposure to light, while its physicochemical and degradation behavior may also be influenced by the chemical environment, including solution pH [35,36]. Although the dye exposure conditions were standardized in the present study, potential interactions between methylene blue and the chemical components of the tested materials cannot be completely excluded. Accordingly, the findings obtained with this methodology should be interpreted as comparative differences in linear dye penetration under the standardized experimental conditions of the present study, rather than as a direct representation of the susceptibility of the tested materials to bacterial recontamination or clinical coronal leakage. Also, the no-IOB control represented an experimental reference condition and did not represent a clinically definitive coronal restoration. Therefore, comparisons involving this group should be interpreted as differences from an unfilled coronal condition rather than as evidence of superiority over a clinically restored tooth. In addition, fully sealed specimens, including sealing of the coronal surface, were not included as negative leakage controls. Although the external surfaces of the specimens were coated with three layers of nail varnish except for the standardized area surrounding the coronal test region, the absence of fully sealed negative controls prevents confirmation that dye penetration occurred exclusively through the intended coronal pathway. Thus, the no-IOB control should be regarded as an experimental comparator rather than a negative leakage control. The present experimental design was intended to isolate differences among IOB materials rather than to assess the complete restorative complex. Nevertheless, the absence of an additional control group in which the IOB was covered by a definitive composite restoration limits assessment of whether an IOB provides additional protection beneath an intact coronal restoration. Future studies incorporating such a control configuration, as well as complementary leakage-assessment methods, may provide a more clinically representative evaluation of the incremental sealing effect of IOBs.

## 5. Conclusions

Within the limitations of this ex vivo study, the type of IOB material significantly influenced coronal microleakage. The conventional universal resin composite (EQ) and both bulk-fill resin composites (EF and FR) demonstrated significantly lower coronal dye penetration than the flowable resin composite (TNF), RMGIC (IS), and no-IOB control. No significant differences were observed among EQ, EF, and FR.

These findings indicate that the conventional universal resin composite and the two bulk-fill resin composite formulations evaluated in the present study demonstrated lower microleakage under the experimental conditions. These results support further investigation of these specific formulations as potential IOB materials. Further in vitro and clinical studies incorporating definitive restorative procedures, restoration-disruption models, mechanical loading, prolonged aging, and alternative leakage assessment methods are warranted to determine the incremental clinical benefit of IOBs.

## Figures and Tables

**Table 1:** Composition, filler content, and manufacturer information of the intra-orifice barrier materials.

Groups	Material	Shade	Manufacturer	Composition	Filler	
					Content by Weight (%)	Content by Volume (%)
Universal composite	Estelite Sigma Quick (EQ)	A2	Tokuyama Dental Corp., Tokyo, Japan	Filler: Supra-nano spherical filler (100–300 nm; average size: 200 nm, SiO_2_–ZrO_2_) Matrix: Bis-GMA, TEGDMA	82%	71%
Flowable composite	Tetric N-Flow (TNF)	A2	Ivoclar Vivadent, Schaan, Liechtenstein	Filler: Barium glass, ytterbium trifluoride, highly dispersed silicon dioxide, and mixed oxide fillers (particle size: 40–3000 nm) Matrix: Bis-GMA, UDMA, and TEGDMA	63.8%	39%
Resin-modified glass ionomer cement	Ionoseal (IS)	A2	VOCO GmbH, Cuxhaven, Germany	Filler: Glass ionomer powder Matrix: UDMA, HEDMA, and TEGDMA	NA	NA
High-viscosity bulk-fill composite	Filtek One Bulk Fill Restorative (FR)	A2	3M ESPE, St. Paul, MN, USA	Filler: Non-agglomerated/non-aggregated 20 nm silica filler, non-agglomerated/non-aggregated 4 to 11 nm zirconia filler, aggregated zirconia/silica cluster filler (comprising 20 nm silica and 4 to 11 nm zirconia particles) and a ytterbium trifluoride filler agglomerate 100 nm particles Matrix: AFM (dynamic stress-relieving monomer), AUDMA, UDMA and 1,12-dodecane-DMA	76.5%	58.5%
**Flowable bulk-fill composite**	Estelite Bulk Fill Flow (EF)	A2	Tokuyama Dental Corp., Tokyo, Japan	Filler: spherical silica, and zirconia (100–300 nm) Matrix: Bis-GMA, Bis-MPEPP, TEGDMA	70%	56%

AFM: addition–fragmentation monomer; AUDMA: aromatic urethane dimethacrylate; Bis-GMA: bisphenol A diglycidyl ether dimethacrylate; Bis-MPEPP: bisphenol A polyethoxy dimethacrylate; DMA: dimethacrylate; HEDMA: 1,6-hexanediol dimethacrylate; NA: not available from the manufacturer; SiO_2_: silicon dioxide; TEGDMA: triethylene glycol dimethacrylate; UDMA: urethane dimethacrylate; ZrO_2_: zirconium dioxide.

**Table 2:** Comparison of mean microleakage values among the experimental groups.

Group	*n*	Microleakage (mm), Mean ± SD	*p*
EQ—Universal resin composite	10	1.85 ± 0.59 ^a^	<0.001 *
TNF—Flowable resin composite	10	4.00 ± 1.37 ^b^	
IS—Resin-modified glass-ionomer cement	10	4.06 ± 0.59 ^b^	
FR—High-viscosity bulk-fill resin composite	10	2.48 ± 1.18 ^a^	
EF—Flowable bulk-fill resin composite	10	2.28 ± 0.91 ^a^	
No-IOB control	10	4.60 ± 1.22 ^b^	

* One-way ANOVA, *p* < 0.001. Values sharing the same superscript letter were not significantly different according to Tukey’s HSD post hoc test (α = 0.05). Abbreviations: EF, Estelite Bulk Fill Flow; EQ, Estelite Sigma Quick; FR, Filtek One Bulk Fill Restorative; IS, Ionoseal; mm, millimeter; SD, standard deviation; TNF, Tetric N-Flow.

## Data Availability

The original contributions presented in this study are included in the article. Further inquiries can be directed to the corresponding author.

## Abbreviations

The following abbreviations are used in this manuscript:

IOB	Intra-orifice barrier
RMGIC	Resin-modified glass ionomer cement
EQ	Estelite Sigma Quick
TNF	Tetric N-Flow
IS	Ionoseal
FR	Filtek One Bulk Fill Restorative
EF	Estelite Bulk Fill Flow
